# Response to the letter to the editor by Kampert et al. entitled “Impact of wearing a facial covering on aerobic exercise capacity in the COVID-19 Era: is it more than a feeling?”

**DOI:** 10.1007/s00392-020-01726-6

**Published:** 2020-08-24

**Authors:** Sven Fikenzer, T. Uhe, D. Lavall, U. Rudolph, R. Falz, M. Busse, P. Hepp, U. Laufs

**Affiliations:** 1 Klinik und Poliklinik für Kardiologie, Universitätsklinikum Leipzig, Liebigstr. 20, 04103 Leipzig, Germany; 2 Institut für Sportmedizin und Prävention, Universität Leipzig, Marschner Str. 29, 04109 Leipzig, Germany; 3 Klinik für Orthopädie, Unfallchirurgie und Plastische Chirurgie, Universitätsklinikum Leipzig, Liebigstr. 20, 04103 Leipzig, Germany

Kampert et al. express questions regarding (1) the measurement of pulmonary function, (2) listing of the data, (3) and a model of blood flow redistribution at peak exercise described by Harms et al. In addition, (4) they comment on the peak metabolic demand of the three conditions.

Ad 1: During CPET, VE sm: −12.0 ± 12.6%, ffpm: −23.1 ± 13.6%, *p* = 0.001, tidal volume sm −9.9 ± 11.3% and ffpm: −14.4 ± 13.0%, *p* = 0.016, inhalation time sm: +12 ± 15%, ffpm: +19 ± 16%, *p* < 0.001, compared to nm.

Ad 2: The complete results are listed in Tables 2 and 3.

Ad 3: Harms et al. (1998) were cited describing possible changes in the distribution of total blood flow with additional work of breathing (e.g. higher breathing resistance) [1].

Ad 4: At maximum load, the metabolic demands were similar in all three conditions. The respiratory exchange ratio (RER) did not differ between the tests (nm 1.13 ± 0.08, sm 1.15 ± 0.09, ffpm 1.13 ± 0.08, one-way ANOVA *p* = 0.596).

A straightforward way to get a feeling for the clear results of the study could be to personally try out the effects of sm and ffpm during exercise.
