# Correction to: Response to the letter to the editor by Kampert et al. entitled “Impact of wearing a facial covering on aerobic exercise capacity in the COVID‑19 Era: is it more than a feeling?”

**DOI:** 10.1007/s00392-021-01966-0

**Published:** 2021-11-19

**Authors:** Sven Fikenzer, T. Uhe, D. Lavall, U. Rudolph, R. Falz, M. Busse, P. Hepp, U. Laufs

**Affiliations:** 1 Klinik und Poliklinik für Kardiologie, Universitätsklinikum Leipzig, Liebigstr. 20, 04103 Leipzig, Germany; 2 Institut für Sportmedizin und Prävention, Universität Leipzig, Marschner Str. 29, 04109 Leipzig, Germany; 3 Klinik für Orthopädie, Unfallchirurgie und Plastische Chirurgie, Universitätsklinikum Leipzig, Liebigstr. 20, 04103 Leipzig, Germany

## Correction to: Clinical Research in Cardiology (2020) 109:1597 10.1007/s00392-020-01726-6

The article “Response to the letter to the editor by Kampert et al. entitled “Impact of wearing a facial covering on aerobic exercise capacity in the COVID‑19 Era: is it more than a feeling?”, written by S. Fikenzer, T. Uhe, D. Lavall, U. Rudolph, R. Falz, M. Busse, P. Hepp and U. Laufs, was originally published Online First without Open Access. After publication in volume 109, issue 12, page 1597 the author decided to opt for Open Choice and to make the article an Open Access publication. Therefore, the copyright of the article has been changed to © The Author(s) 2020 and the article is forthwith distributed under the terms of the Creative Commons Attribution 4.0 International License, which permits use, sharing, adaptation, distribution and reproduction in any medium or format, as long as you give appropriate credit to the original author(s) and the source, provide a link to the Creative Commons licence, and indicate if changes were made.

The images or other third party material in this article are included in the article’s Creative Commons licence, unless indicated otherwise in a credit line to the material. If material is not included in the article’s Creative Commons licence and your intended use is not permitted by statutory regulation or exceeds the permitted use, you will need to obtain permission directly from the copyright holder.

To view a copy of this licence, visit http://creativecommons.org/licenses/by/4.0/.

The original article has been corrected.

